# Association between infrapatellar fat pad ultrasound elasticity and anterior knee pain in patients with knee osteoarthritis

**DOI:** 10.1038/s41598-023-47459-0

**Published:** 2023-11-16

**Authors:** Yoshinori Satake, Masashi Izumi, Koji Aso, Masahiko Ikeuchi

**Affiliations:** Department of Orthopaedic Surgery, Kochi Medical School, Kochi University, Kohasu, Okoh-Cho, Nankoku-City, Kochi 783-8505 Japan

**Keywords:** Diseases, Medical research

## Abstract

This study investigates whether infrapatellar fat pad (IPFP) elasticity is associated with anterior knee pain in patients with knee osteoarthritis (KOA). The IPFP elasticity of 97 patients with KOA (Kellgren and Lawrence [KL] grades of the femorotibial and patellofemoral joints ≥ 2 and ≤ 2, respectively), aged 46–86 years, was evaluated via shear wave speed using ultrasound elastography. The patients were divided into two groups according to the presence or absence of anterior knee pain. Univariate analyses were used to compare patient age, sex, femorotibial KL grade, magnetic resonance imaging findings (Hoffa, effusion synovitis, bone marrow lesion scores, and IPFP size), and IPFP elasticity between the groups. Multivariate logistic regression analyses were subsequently performed using selected explanatory variables. IPFP elasticity was found to be associated with anterior knee pain in the univariate (p = 0.007) and multivariate (odds ratio: 61.12, 95% CI 1.95–1920.66; p = 0.019) analyses. Anterior knee pain is strongly associated with stiffer IPFPs regardless of the femorotibial KL grade, suggesting that ultrasound elastography is useful for the diagnosis of painful IPFP in patients with KOA.

## Introduction

Knee osteoarthritis (KOA) is a progressive, degenerative disease and one of the most common causes of physical disability^1^. Symptomatic KOA occurs in 20–30% of the elderly population aged ≥ 65 years^2,3^, and its prevalence is increasing worldwide, partly due to the aging population^4^. Pain is the most common reason that patients request medical care; however, due to the inability to halt the progression of osteoarthritis (OA), treatments focus on relieving pain and maintaining joint function^5^.

KOA is considered to be a whole-joint disease involving all structures in the knee joint. Recent studies have provided deeper insights into the pathology of OA at each structure, including the cartilage, synovium, subchondral bone, and muscles^6^. Traditionally, radiography was used to assess the severity of KOA, though discordance between the radiographic grading of KOA and the severity of pain has been reported^7^. Therefore, the use of magnetic resonance imaging (MRI) to evaluate the knee joint has increased, and correlations between knee pain severity and MRI findings such as effusion synovitis and bone marrow lesions (BMLs) have been reported^8^.

Patients with KOA often report anterior knee pain. MRI findings of patellofemoral (PF) osteophytes, effusion synovitis, and infrapatellar synovitis are associated with anterior knee pain^9^. Although anterior knee pain is induced by several factors^10^, its pathophysiology remains unclear.

The infrapatellar fat pad (IPFP) is an intraarticular and extra-synovial adipose structure of richly innervated tissue in the knee joint^11^. The IPFP increases the articular congruity, lubricating the joint and serving as a protective cushion to the joint^6^. The IPFP may act as an endocrine organ by secreting several catabolic cytokines^12,13^. Moreover, the IPFP is a known source of anterior knee pain^11^. The role of the IPFP in the knee joint is complicated and not fully understood.

Recent findings suggest that the IPFP plays a role in the pathogenesis of KOA^14^. Along with synovitis, IPFP fibrosis is one of the most common findings in patients with KOA^15^. Due to advances in ultrasound elastography technology, the elasticity of soft tissues can be evaluated. Elastography has been clinically utilized to assess several diseases, including breast cancer, chronic hepatitis^16^, rotator cuff tears^17^, and Achilles tendinopathy^18^. However, despite the fact that OA pathology involves soft tissue around the joint, IPFP elasticity has rarely been assessed. IPFP fibrosis may be actively involved in OA progression and pain generation^19,20^. As the use of ultrasound elastography has been recommended for the assessment of liver fibrosis^21^, elastography of the IPFP in patients with KOA was conducted to evaluate the elasticity, which presumably reflects fibrosis.

Therefore, this study clarifies the elastic properties of the IPFP in patients with symptomatic KOA, focusing on radiological OA stages and anterior knee pain. It was hypothesized that IPFP elasticity is associated with anterior knee pain, regardless of the stage of OA.

## Methods

### Patients

This cross-sectional study included a convenience sample of 120 patients diagnosed with KOA (Kellgren and Lawrence [KL] grade ≥ 2)^22^ who visited our hospital between July 2020 and December 2021. Patients with systemic inflammatory diseases (such as rheumatoid arthritis), mental handicaps or psychiatric conditions, or a history of surgery on the affected knee were excluded from the study. Patients with PF joint OA (KL grade of ≥ 3) were also excluded to minimize the effect of PF joint OA on the study results. This study was approved by the Ethical Review Board of Kochi Medical School (approval number 2020-11). A verbal explanation of the study was provided to each patient, and written informed consent was obtained from each patient prior to their participation in the study. This study was conducted in compliance with the Declaration of Helsinki.

### Outline of the experimental protocol

On the first visit, the patients’ demographic data were assessed and knee radiography and pressure pain threshold (PPT) measurements of the IPFP were performed. Knee MRI and ultrasound elastography of the IPFP were conducted on a different day for the patients’ convenience. Partial excision biopsies of the IPFP were performed in patients who subsequently underwent knee surgery.

### Demographic data

**Figure 1:**
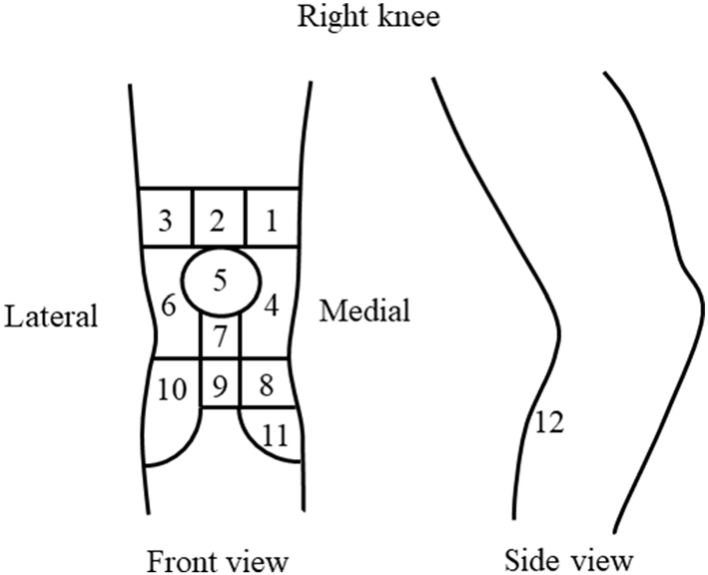
Knee pain map. A knee pain map of the right knee is shown. Patient-reported pain in areas 5 (patella) or 7 (patellar ligament) while walking is categorized as anterior knee pain.

Patient demographic data (sex, age, height, and weight) and the clinical characteristics of the affected knee were assessed. Knee pain maps have been used for the evaluation of anterior knee pain^23^. The knee pain map developed by Hill et al.^24^, which includes 12 areas separated by grid lines, was used in this study (Fig. 1). Area 1 represents the anterior superomedial aspect of the knee, while area 2 represents the anterior superior aspect of the knee. The anterior superolateral aspect of the knee is represented in area 3, the anterior medial aspect in area 4, the patella in area 5, and the anterior lateral aspect in area 6. Area 7 represents the patellar ligament, area 8 represents the pes anserinus, and area 9 represents the tibial tuberosity. The anterior inferolateral aspect of the knee is represented by area 10, the anterior inferomedial aspect by area 11, and the popliteal aspect by area 12^25^. The patella and patellar ligament (areas 5 and 7, respectively) were directly palpated and the patients were shown the locations of these areas on the knee pain map. Then, the patients walked at a comfortable speed and identified the painful areas of the knee while walking. If areas 5 or 7 were reported to be painful, the pain was categorized as anterior knee pain. Patients were subsequently divided into two groups based on the presence or absence of anterior knee pain.

### PPT measurement

To quantitatively assess IPFP tenderness, one author (YS) measured the PPT 1 cm below the inferior pole of the patella using a digital hand algometer (SDMEIC Electronics, Solna, Sweden) with a 1 cm^2^ probe. Pressure was applied at a rate of 30 kPa/s until the patient-reported pain. The PPT was measured five times at 20-s intervals, and the average of the middle three values was included in the study analyses. The intra-rater test–retest reliability was confirmed using data from 10 volunteers (intraclass correlation coefficient [ICC] (1, K): 0.97).

### Imaging findings

The knee was radiographically examined in a standing position on the anteroposterior view, as well as on the lateral and skyline views. The severities of OA in the femorotibial (FT) and PF joints were assessed using the KL grade (0, none; 1, doubtful joint space narrowing with possible osteophytes; 2, possible joint space narrowing with definite osteophytes; 3, definite joint space narrowing, moderate osteophytes, some sclerosis; and 4, severe joint space narrowing, large osteophytes, marked sclerosis, bone deformities). Affected knees were scanned using 3 T MRI. The following image sequences were used: a T1-weighted three-dimensional (3D) spoiled gradient recall (SPGR) acquisition with fat saturation in the steady state (flip angle: 30°, repetition time: 16–18 ms, echo time: 3.3–4.7 ms, field of view: 16 cm, slice gap: 0 mm, slice thickness: 1.5 mm, sagittal images obtained at a slice thickness of 1.5 mm without an interslice gap) and a T2-weighted two-dimensional fast spin echo with fat suppression (flip angle: 90–142°, repetition time: 2500 ms, echo time: 100 ms, field of view: 16 cm, axial, sagittal, and coronal images obtained at a slice thickness of 4 mm with an interslice gap of 1 mm).

The Hoffa score, assessed using the MRI Osteoarthritis Knee Score^26^, was graded from 0 to 3 based on the degree of IPFP hyperintensity (0, normal; 1, mild; 2, moderate; and 3, severe). The effusion synovitis and BML scores were assessed using the whole-organ MRI score^27^. The effusion synovitis score was graded from 0 to 3 based on the estimated maximal distention of the synovial cavity (0, normal; 1, < 33% of the maximum potential distention; 2, 33–66% of the maximum potential distention; and 3, > 66% of the maximum potential distention). The BMLs were graded from 0–3 at all 15 areas around the knee joint based on the degree of bone hyperintensity (0, none; 1, < 25%; 2, 25–50%; and 3, > 50%). The BML score was calculated as the sum of the BML grades. All images were evaluated by a single observer (YS).

The IPFP area was measured by manually delineating the contour of the IPFP on each SPGR image using the software program OsiriX Lite (Pixmeo SARL, Bernex, Switzerland). A 3D reconstruction of the IPFP was created, and the IPFP volume was automatically calculated. A single observer (YS) graded the IPFP volume. The IPFP size was evaluated by dividing the IPFP volume by the body weight of each patient to adjust for the physique.

### Ultrasound shear wave elastography (SWE)

Point SWE measurement of the IPFP was performed by one author (YS) using ultrasonography (ARIETTA E70; FUJIFILM Healthcare Corporation, Tokyo, Japan) with a C251 convex probe (1–5 MHz; FUJIFILM Healthcare Corporation). All patients were examined in the supine position at 20° of knee flexion, achieved using a rolled towel under the knee. B-mode axial images of the IPFP were visualized by placing the probe over the patellar ligament. The 10 × 15 mm^2^ region of interest^28^ was placed in the middle of the axial sectional view at the midpoint level of the IPFP. The IPFP in the region of interest was vibrated via acoustic radiation force impulses, and the shear wave speed was automatically measured. The depth of the region of interest was set at less than 6 cm to avoid acoustic radiation force impulse attenuation^21^. The elastography measurements were performed only when the percentage of the net amount of the effective shear wave velocity (VsN) was ≥ 50%. Measurements were performed five times, and the average of the middle three values was used in the analyses. The inter-rater test–retest reliability was confirmed using data from five participants (ICC (1, K): 0.83).

### Histological IPFP fibrosis

Partial excision biopsies were performed at the central distal third of the IPFP in patients who underwent knee surgery after the study assessments. The samples were fixed in 10% formalin and embedded in paraffin, and three nonconsecutive sections containing the synovial membrane were obtained from the paraffin-embedded samples and stained with Masson’s trichrome. The lesion with the most extensive fibrosis among the three sections was detected using a microscope (BZ-X800; Keyence, Osaka, Japan) at 40 × magnification. IPFP fibrosis was evaluated using the ImageJ Fiji package^29^. The image of the lesion with the most extensive fibrosis was split into RGB channels, and image calculations were performed (blue image minus red image). The black area representing fibrosis was measured and expressed as a percentage.

### Statistical analysis

Spearman’s rank correlation coefficient was used to compare the IPFP characteristics (size and elasticity) and the KL grade of the FT joint. Univariate analyses among the two groups—divided by the presence or absence of anterior knee pain—were conducted to compare patient age, sex, KL grade of the FT joint, Hoffa score, effusion synovitis score, BML score, IPFP size, and shear wave speed. The Fisher’s exact test and the Mann–Whitney U test were used because several parameters were non-normally distributed variables. A logistic regression analysis was conducted using factors with p-values ≤ 0.2 in the univariate analyses as explanatory variables after adjustment for sex and age. The KL grade is a widely used classification for KOA severity and has clinical significance; therefore, the logistic regression analysis was performed on the model with the KL grade added as an explanatory factor.

The correlation between the percentage of IPFP fibrosis and shear wave speed was examined using Pearson’s correlation coefficient to evaluate the reliability of SWE. The normality of the data was assessed via a visual inspection of the normality plot. Statistical analyses were performed using Bell Curve for Excel (Social Survey Research Information Co., Ltd., Tokyo, Japan) and EZR (Saitama Medical Center, Jichi Medical University, Saitama, Japan). A p-value < 0.05 was considered statistically significant.

## Results

### Patient characteristics

After the exclusion of 23 patients due to MRI examination difficulty (n = 1), IPFP compression by a ganglion (n = 1), mental handicap (n = 2), evaluation by 1.5 T MRI (n = 2), poor physical condition (n = 3), and PF joint KL grade ≥ 3 (n = 14), 97 patients were included in the final analysis. The SIGNA Architect 3.0 T MRI system (GE Healthcare, Chicago, IL, US) was used to evaluate 73 knees, and the Ingenia 3.0 T MRI system (Philips, Amsterdam, NL) was used to evaluate 24 knees. Seventy-three patients were women, and the mean patient age was 71.7 years (range 46–86 years) (Table 1). Patients with a KL grade of the FT joint of 4 had higher shear wave speeds (p < 0.001) than patients with other KL grades (Table 2).

**Table 1:** Patient characteristics (n = 97).

Age (years)	71.7 ± 8.7
Female sex	73 (75.3)
Height (cm)	154.7 ± 8.8
Weight (kg)	64.8 ± 15.0
BMI (kg/m^2^)	27.0 ± 5.0
Right side	52 (53.6)
KL grade (FT joint)	
2	21 (21.6)
3	36 (37.1)
4	40 (41.2)
KL grade (PF joint)	
0	3 (3.1)
1	18 (18.6)
2	76 (78.4)
Hoffa score	1.3 ± 0.5
Effusion synovitis score	1.3 ± 0.7
BML score	5.9 ± 4.3
IPFP volume (cm^3^)	22.1 ± 6.3
IPFP size (cm^3^/kg)	0.35 ± 0.08
Shear wave speed (m/s)	1.38 ± 0.17

Data are presented as mean ± standard deviation or number (percentage).

*BMI* body mass index, *KL* Kellgren and Lawrence, *FT* femorotibial, *PF* patellofemoral, *BML* bone marrow lesion, *IPFP* infrapatellar fat pad.

**Table 2:** Patient characteristics according to KL grade of FT joint (n = 97).

	KL grade of FT joint			P-value
	2	3	4	
Participants	21	36	40	
Age (years)	66.2 ± 9.6	72.6 ± 7.8*	73.8 ± 7.9*	0.010
Female sex	16 (76.2)	23 (63.9)	34 (85)	0.104
Height (cm)	157.0 ± 8.0	156.5 ± 8.6	151.7 ± 8.6*^†^	0.010
Weight (kg)	65.7 ± 18.8	64.1 ± 14.8	65.1 ± 13.3	0.733
BMI (kg/m^2^)	26.3 ± 5.4	26.0 ± 4.4	28.2 ± 5.2	0.092
Right side	13 (61.9)	18 (50)	21 (52.5)	0.700
KL grade (PF joint)				< 0.001
0	3 (14.3)	0 (0)	0 (0)	
1	11 (52.4)	6 (16.7)	1 (2.5)	
2	7 (33.3)	30 (83.3)	39 (97.5)	
Hoffa score	1.0 ± 0.4	1.3 ± 0.5	1.5 ± 0.6*	0.029
Effusion synovitis score	1.0 ± 0.7	1.2 ± 0.7	1.6 ± 0.6*^†^	0.006
BML score	2.3 ± 1.7	5.6 ± 3.5*	8.1 ± 4.7*	< 0.001
IPFP volume (cm^3^)	24.0 ± 7.0	23.1 ± 6.7	20.3 ± 5.0	0.071
IPFP size (cm^3^/kg)	0.37 ± 0.08	0.36 ± 0.07	0.32 ± 0.07^†^	0.018
Shear wave speed (m/s)	1.30 ± 0.15	1.33 ± 0.12	1.48 ± 0.17*^†^	< 0.001

Data are presented as mean ± standard deviation or number (percentage).

*KL* Kellgren and Lawrence, *FT* femorotibial, *BMI* body mass index, *PF* patellofemoral, *BML* bone marrow lesion, *IPFP* infrapatellar fat pad.

Fisher’s exact, Kruskal–Wallis, and Steel–Dwass test: *P < 0.05 compared with KL grade 2; ^†^P < 0.05 compared with KL grade 3.

### Association of IPFP size and elasticity with radiographic severity

The IPFP was significantly smaller (r = − 0.28; p = 0.006) and stiffer (r = 0.45; p < 0.001) as the KL grade severity of the FT joint increased (Spearman’s rank correlation coefficient).

### Predisposing factors associated with anterior knee pain

Forty-one patients (42.3%) had anterior knee pain and exhibited significantly lower PPTs than patients without anterior knee pain (mean PPT: 419.5 ± 146.5 kPa vs. 498.9 ± 144.5 kPa; p = 0.006; Mann–Whitney U test). The shear wave speed was significantly associated with anterior knee pain in the univariate analyses (Table 3). Logistic regression analyses indicated that shear wave speed was the only significant explanatory variable of anterior knee pain (Table 4). The shear wave speed was also associated with PPT (r = − 0.20; p = 0.046; Pearson’s correlation analysis).

**Table 3:** Univariate analyses for predisposing factors associated with anterior knee pain (n = 97).

Parameter	Anterior knee pain		
	Positive (n = 41)	Negative (n = 56)	P-value
Demographic data			
Age (years)	71.3 ± 10.5	71.9 ± 7.1	0.818
Female sex	35 (85.4)	38 (67.9)	0.059
Radiograph			
KL grade of FT joint			
2	7 (17.1)	14 (25)	0.233
3	13 (31.7)	23 (41.1)	
4	21 (51.2)	19 (33.9)	
MRI			
Hoffa score			
0	0 (0)	2 (3.6)	0.270
1	28 (68.3)	37 (66.1)	
2	11 (26.8)	17 (30.4)	
3	2 (4.9)	0 (0)	
Effusion synovitis score			
0	2 (4.9)	8 (14.3)	0.298
1	19 (46.3)	29 (51.8)	
2	19 (46.3)	18 (32.1)	
3	1 (2.4)	1 (1.8)	
BML score			
Total	7.0 ± 4.7	5.1 ± 3.9	0.060
PF joint (4 areas)	1.3 ± 1.2	1.0 ± 1.0	0.289
FT joint (11 areas)	5.7 ± 4.1	4.1 ± 3.3	0.058
IPFP size (cm^3^/kg)	0.33 ± 0.07	0.36 ± 0.08	0.078
Elastography			
Shear wave speed(m/s)	1.45 ± 0.19	1.34 ± 0.14	0.007

Data are presented as mean ± standard deviation or number (percentage).

Fisher’s exact and the Mann–Whitney U test were used.

*KL* Kellgren and Lawrence, *FT* femorotibial, *MRI* magnetic resonance imaging, *BML* bone marrow lesion, *PF* patellofemoral, *IPFP* infrapatellar fat pad.

**Table 4:** Logistic regression analysis for predisposing factors associated with anterior knee pain (n = 97).

Predisposing factor	OR	95% CI	P-value
KL grade of FT joint	0.88	0.42–1.82	0.726
BML score	1.07	0.93–1.23	0.337
IPFP size	0.01	0.000–11.00	0.196
Shear wave speed	61.12	1.95–1920.66	0.019

Data are adjusted for sex and age.

*OR* odds ratio, *CI* confidence interval, *KL* Kellgren and Lawrence, *FT* femorotibial, *BML* bone marrow lesion, *IPFP* infrapatellar fat pad.

### Association between the percentage of maximal fibrotic lesions and shear wave speed

**Figure 2:**
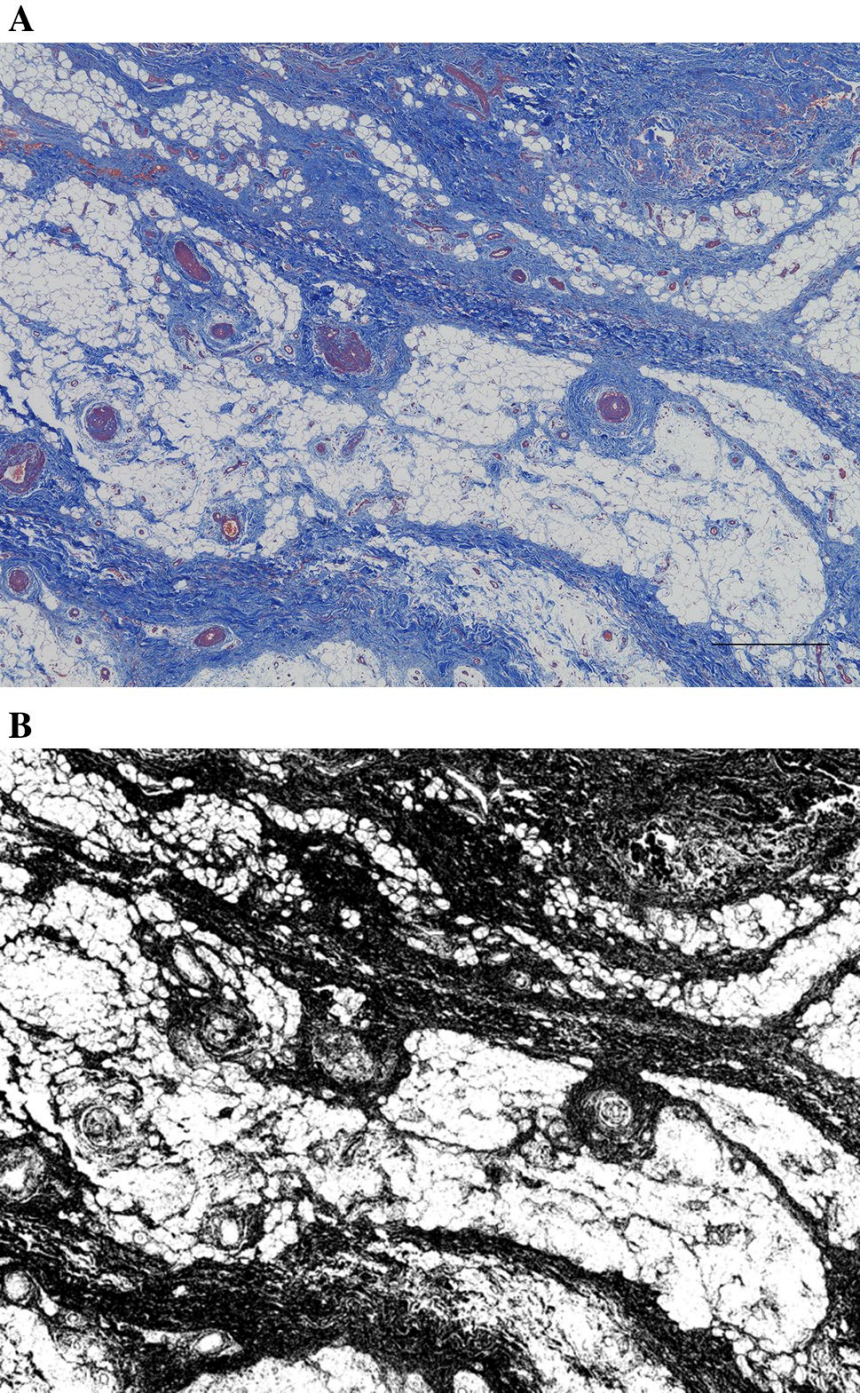
Histological infrapatellar fat pad fibrosis evaluation. (**A**) The infrapatellar fat pad (IPFP) lesion with the most extensive fibrosis of that in three nonconsecutive slice sections is detected in each sample using a microscope at 40 × magnification (scale bar = 500 µm). (**B**) The image of the IPFP lesion with the most extensive fibrosis is split into RGB channels. The black area representing the fibrosis is measured and expressed as the percentage. Image calculations are conducted using ImageJ Fiji package.

**Figure 3:**
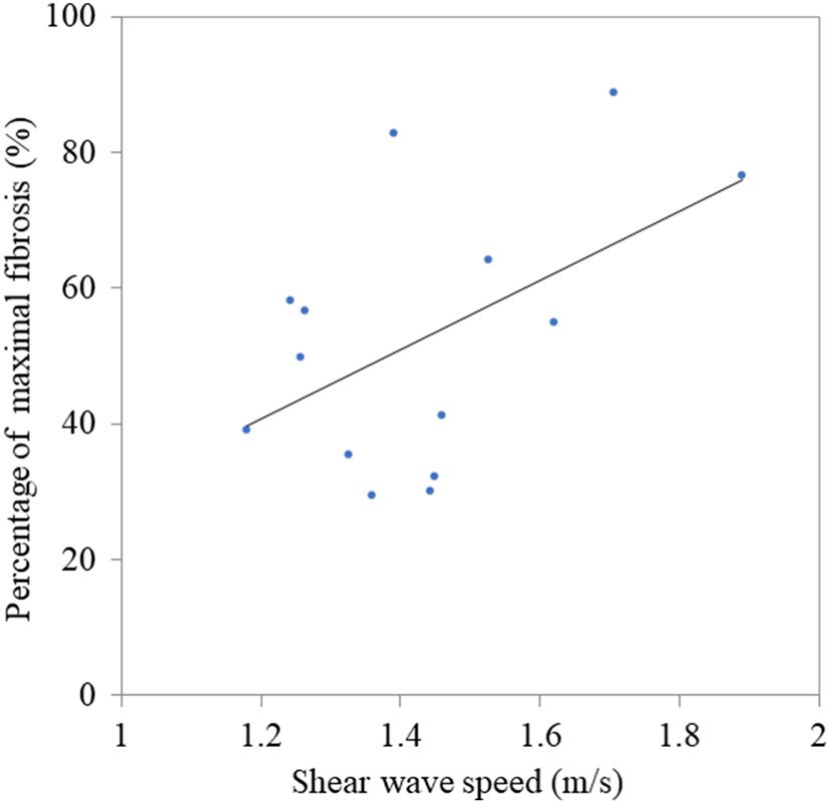
Association between the percentage of maximal fibrotic lesions and shear wave speed in the infrapatellar fat pad. The correlation between the percentage of maximal fibrosis and the shear wave speed is evaluated using 14 infrapatellar fat pad specimens (correlation coefficient: 0.51; p = 0.062).

Fourteen IPFP samples obtained from 14 patients were histologically evaluated. The mean patient age was 73.6 years (range 59–84 years), and the KL grades of the FT joint were 3 (n = 3) and 4 (n = 11). The mean percentage of fibrosis was 52.8% (range 29.5–88.8%) (Fig. 2). The percentage of maximal fibrosis tended to increase as the shear wave speed increased, though the correlation was not significant (r = 0.51; p = 0.062; Pearson’s correlation analysis) (Fig. 3).

## Discussion

In this study, smaller and stiffer IPFPs were associated with radiographic evidence of femorotibial osteoarthritis (FT-OA) progression, and patients with anterior knee pain had stiffer IPFPs regardless of the stage of FT-OA. This is the first study to evaluate the relationship between IPFP elasticity and anterior knee pain.

During SWE, the target tissue is vibrated using acoustic radiation force impulses, and the generated shear wave propagation speed is measured^16^. SWE provides quantitative evaluations and is less dependent on the examiner than other ultrasound elastography methods. In contrast, SWE results may vary due to the heterogeneous acoustic properties of tissues and refraction of the shear wave^16^. In this study, the IPFP was evaluated at a unified position and axis to achieve an accurate and consistent examination. In addition, only values with a VsN ≥ 50% were considered reliable. The VsN is a reliability index, and a cutoff value of 50% is widely utilized for evaluating the shear wave speed when staging liver fibrosis^28,30^. Although no significant correlation was observed between the percentage of maximal fibrotic lesions and shear wave speed, the percentage of fibrosis tended to increase as the shear wave speed increased. Takahashi et al.^31^ reported a correlation between liver elasticity evaluated by ultrasound elastography and histological liver fibrosis, which partly supports the results of the current study.

The 3D size of the IPFP was previously evaluated by measuring its maximum area on a midsagittal MRI image^32^. In this study, the IPFP volume shown on a 3D-reconstructed image was used for a more accurate assessment of the IPFP size. Furthermore, the IPFP size was evaluated as volume/weight based on the correlation between the IPFP volume and body weight^33^. A smaller IPFP is associated with the radiographic progression of FT-OA. Several studies have compared the IPFP volume of patients with KOA to healthy controls^34,35^. Fontanella et al. reported a decrease in the IPFP volume as KOA progressed, though the differences in IPFP volume were not significant^35^. The differences between the results of the current study and previous findings^35^ may be owing to the relatively larger number of patients, weight adjustment for the IPFP volume, and high proportion of end-stage OA observed in the current study. The IPFP was found to be stiffer as radiographic progression of FT-OA was observed. Favero et al. reported that the thickness of the interlobular septa and fibrosis in the adjacent synovial membrane were higher in patients with KOA than in healthy controls^15^. The shear wave speed was estimated to reflect these IPFP changes; therefore, the results of the current study are consistent with previous findings.

MRI provides semiquantitative data regarding the IPFP in studies regarding knee pain. The inconsistent findings of previous studies^36–38^ may be due to differences in pain measurements or KOA severity. Hence, anterior knee pain associated with the IPFP was evaluated in this study. Subjective anterior knee pain evaluations were supported by quantitative PPT data. IPFP fibrosis is common in patients with KOA, suggesting that IPFP fibrosis plays an essential role in OA pathophysiology. Quantitative assessments of IPFP fibrosis via MRI are difficult. Therefore, SWE was used in this study. The combination of ultrasound elastography and MRI provides a multifaceted evaluation of the IPFP.

Patients with KOA and anterior knee pain have stiffer IPFPs in this study; however, anterior knee pain is not correlated with IPFP size. Onuma et al. reported that irreversible IPFP fibrosis resulted in sprouting of calcitonin gene-related peptide-positive nerve fibers and prolonged pain in rats^20^. Similarly, the results of the current study suggest that IPFP inflammation induces changes in the nerve endings, and concomitant fibrosis was inferred by SWE, resulting in a significant association between the shear wave speed and anterior knee pain. Furthermore, the surface of the IPFP adjacent to the synovium had severe fibrosis in several specimens, synovitis can be a primary cause of IPFP fibrosis. Therefore, the IPFP evaluation using ultrasound elastography can help diagnose painful IPFP in patients with KOA. A recent study that included contrast-enhanced MRI demonstrated that the increased IPFP synovium volume was significantly associated with knee pain^39^. Ultrasound is more convenient and cost-effective than MRI and is also noninvasive. The results of the current study will contribute to the development of treatments for painful IPFP in patients with KOA.

This study has several limitations. First, the cross-sectional nature of this study precludes any inference regarding the causality between IPFP characteristics and anterior knee pain. Nevertheless, a significant association between IPFP elasticity and anterior knee pain was identified in this study, suggesting the possibility that the causality be resolved in the future. Second, many patients had a KL of grade 3 or 4; therefore, these results may not apply to patients with early-stage KOA. Third, the effects of other disorders and treatments that may affect knee pain, such as spinal disorders and painkillers, cannot be ruled out. Fourth, other potential pain factors, such as sensitization and psychosocial factors, were not assessed in this study. Fifth, the severity of knee pain was not assessed. It is likely that stiffer IPFPs are found in severely painful knees. In addition, the same MRI model was not used to evaluate all of the patients. However, no significant differences in patient sex, age, height, or weight were noted between the groups evaluated using each MRI system. Last, a histological evaluation was not performed for all patients. Despite these limitations, this study provides useful insights regarding the IPFP elasticity and anterior knee pain in patients with KOA.

In conclusion, patients with KOA and anterior knee pain have stiffer IPFPs regardless of FT-OA grade, indicating that ultrasound elastography is useful for the diagnosis of painful IPFP in patients with KOA.

## Data Availability

Additional data is available upon reasonable request to the corresponding author.

## References

[1] Guccione AA (1994). The effects of specific medical conditions on the functional limitations of elders in the Framingham Study. Am. J. Public Health..

[2] Mannoni A (2003). Epidemiological profile of symptomatic osteoarthritis in older adults: A population based study in Dicomano, Italy. Ann. Rheum. Dis..

[3] Andrianakos AA (2006). Prevalence of symptomatic knee, hand, and hip osteoarthritis in Greece. The ESORDIG study. J. Rheumatol..

[4] Leveille SG (2004). Musculoskeletal aging. Curr. Opin. Rheumatol..

[5] Imamura M (2008). Impact of nervous system hyperalgesia on pain, disability, and quality of life in patients with knee osteoarthritis: A controlled analysis. Arthritis Rheum..

[6] Zeng N, Yan ZP, Chen XY, Ni GX (2020). Infrapatellar fat pad and knee osteoarthritis. Aging Dis..

[7] Bedson J, Croft PR (2008). The discordance between clinical and radiographic knee osteoarthritis: A systematic search and summary of the literature. BMC Musculoskelet. Disord..

[8] Yusuf E, Kortekaas MC, Watt I, Huizinga TW, Kloppenburg M (2011). Do knee abnormalities visualised on MRI explain knee pain in knee osteoarthritis? A systematic review. Ann. Rheum. Dis..

[9] Macri EM (2022). Magnetic resonance imaging-defined osteoarthritis features and anterior knee pain in individuals with, or at risk for, knee osteoarthritis: A multicenter study on osteoarthritis. Arthritis Care Res. (Hoboken).

[10] Werner S (2014). Anterior knee pain: An update of physical therapy. Knee Surg. Sports Traumatol. Arthrosc..

[11] Bennell K, Hodges P, Mellor R, Bexander C, Souvlis T (2004). The nature of anterior knee pain following injection of hypertonic saline into the infrapatellar fat pad. J. Orthop. Res..

[12] Kershaw EE, Flier JS (2004). Adipose tissue as an endocrine organ. J. Clin. Endocrinol. Metab..

[13] Chang J (2018). Systemic and local adipose tissue in knee osteoarthritis. Osteoarthr. Cartil..

[14] Clockaerts S (2010). The infrapatellar fat pad should be considered as an active osteoarthritic joint tissue: A narrative review. Osteoarthr. Cartil..

[15] Favero M (2017). Infrapatellar fat pad features in osteoarthritis: A histopathological and molecular study. Rheumatology (Oxford).

[16] Shiina T (2013). JSUM ultrasound elastography practice guidelines: Basics and terminology. J. Med. Ultrason..

[17] Tudisco C (2015). Tendon quality in small unilateral supraspinatus tendon tears. Real-time sonoelastography correlates with clinical findings. Knee Surg. Sports Traumatol. Arthrosc..

[18] De Zordo T (2010). Real-time sonoelastography: Findings in patients with symptomatic achilles tendons and comparison to healthy volunteers. Ultraschall Med..

[19] Clements KM (2009). Cellular and histopathological changes in the infrapatellar fat pad in the monoiodoacetate model of osteoarthritis pain. Osteoarthr. Cartil..

[20] Onuma H (2020). Fibrotic changes in the infrapatellar fat pad induce new vessel formation and sensory nerve fiber endings that associate prolonged pain. J. Orthop. Res..

[21] Barr RG (2016). Elastography assessment of liver fibrosis: Society of radiologists in ultrasound consensus conference statement. Ultrasound Q..

[22] Kellgren JH, Lawrence JS (1957). Radiological assessment of osteo-arthrosis. Ann. Rheum. Dis..

[23] Macri EM (2020). Relation of patellofemoral joint alignment, morphology, and radiographic osteoarthritis to frequent anterior knee pain: Data from the multicenter osteoarthritis study. Arthritis Care Res. (Hoboken).

[24] Hill CL (2003). Periarticular lesions detected on magnetic resonance imaging: Prevalence in knees with and without symptoms. Arthritis Rheum..

[25] Ikeuchi M, Izumi M, Aso K, Sugimura N, Tani T (2013). Clinical characteristics of pain originating from intra-articular structures of the knee joint in patients with medial knee osteoarthritis. Springerplus.

[26] Hunter DJ (2011). Evolution of semi-quantitative whole joint assessment of knee OA: MOAKS (MRI osteoarthritis knee score). Osteoarthr. Cartil..

[27] Peterfy CG (2004). Whole-organ magnetic resonance imaging score (WORMS) of the knee in osteoarthritis. Osteoarthr. Cartil..

[28] Yada N (2015). A newly developed shear wave elastography modality: With a unique reliability index. Oncology.

[29] Schindelin J (2012). Fiji: An open-source platform for biological-image analysis. Nat. Methods.

[30] Ferraioli G (2017). Ruling-in and ruling-out significant fibrosis and cirrhosis in patients with chronic hepatitis C using a shear wave measurement method. J. Gastrointest. Liver Dis..

[31] Takahashi H (2010). Evaluation of acoustic radiation force impulse elastography for fibrosis staging of chronic liver disease: A pilot study. Liver Int..

[32] Han W (2014). Infrapatellar fat pad in the knee: Is local fat good or bad for knee osteoarthritis?. Arthritis Res. Ther..

[33] Diepold J (2015). Sex-differences of the healthy infra-patellar (Hoffa) fat pad in relation to intermuscular and subcutaneous fat content–data from the Osteoarthritis Initiative. Ann. Anat..

[34] Chuckpaiwong B, Charles HC, Kraus VB, Guilak F, Nunley JA (2010). Age-associated increases in the size of the infrapatellar fat pad in knee osteoarthritis as measured by 3T MRI. J. Orthop. Res..

[35] Fontanella CG (2019). Quantitative MRI analysis of infrapatellar and suprapatellar fat pads in normal controls, moderate and end-stage osteoarthritis. Ann. Anat..

[36] Ballegaard C (2014). Knee pain and inflammation in the infrapatellar fat pad estimated by conventional and dynamic contrast-enhanced magnetic resonance imaging in obese patients with osteoarthritis: A cross-sectional study. Osteoarthr. Cartil..

[37] Steidle-Kloc E (2018). Relationship between knee pain and infrapatellar fat pad morphology: A within- and between-person analysis from the osteoarthritis initiative. Arthritis Care Res. (Hoboken).

[38] Carotti M, Salaffi F, Di Carlo M, Giovagnoni A (2017). Relationship between magnetic resonance imaging findings, radiological grading, psychological distress and pain in patients with symptomatic knee osteoarthritis. Radiol. Med..

[39] Perry TA, Yang X, van Santen J, Arden NK, Kluzek S (2021). Quantitative and semi-quantitative assessment of synovitis on MRI and the relationship with symptoms in symptomatic knee osteoarthritis. Rheumatology (Oxford).

